# Proof of Aerobically Autoxidized Self-Charge Concept Based on Single Catechol-Enriched Carbon Cathode Material

**DOI:** 10.1007/s40820-023-01283-3

**Published:** 2023-12-20

**Authors:** Junyan Wang, Wanchun Guo, Kesong Tian, Xinta Li, Xinyu Wang, Panhua Li, Yu Zhang, Bosen Zhang, Biao Zhang, Shuhu Liu, Xueai Li, Zhaopeng Xu, Junjie Xu, Haiyan Wang, Yanglong Hou

**Affiliations:** 1 State Key Laboratory of Metastable Materials Science and Technology, Hebei Key Laboratory of Heavy Metal Deep-Remediation in Water and Resource Reuse, School of Environmental and Chemical Engineering, Yanshan University, Qinhuangdao, 066004 People’s Republic of China; 2 Beijing Key Laboratory for Magnetoelectric Materials and Devices, Beijing Innovation Centre for Engineering Science and Advanced Technology, School of Materials Science and Engineering, Peking University, Beijing, 100871 People’s Republic of China; 3 Institute of High Energy Physics, Chinese Academy of Sciences, Beijing, 100049 People’s Republic of China; 4 Xi’an Rare Metal Materials Institute Co. Ltd, Xi’an, 710016 People’s Republic of China

**Keywords:** Carbon material, Oxygen functionality, Air oxidation self-charge

## Abstract

**Highlights:**

- An air-breathing chemical self-charge concept of oxygen-enriched carbon cathode.
- The oxygen-enriched carbon material with abundant catechol groups.
- Rapid air-oxidation chemical self-charge of catechol groups.

**Abstract:**

The self-charging concept has drawn considerable attention due to its excellent ability to achieve environmental energy harvesting, conversion and storage without an external power supply. However, most self-charging designs assembled by multiple energy harvesting, conversion and storage materials increase the energy transfer loss; the environmental energy supply is generally limited by climate and meteorological conditions, hindering the potential application of these self-powered devices to be available at all times. Based on aerobic autoxidation of catechol, which is similar to the electrochemical oxidation of the catechol groups on the carbon materials under an electrical charge, we proposed an air-breathing chemical self-charge concept based on the aerobic autoxidation of catechol groups on oxygen-enriched carbon materials to *ortho*-quinone groups. Energy harvesting, conversion and storage functions could be integrated on a single carbon material to avoid the energy transfer loss among the different materials. Moreover, the assembled Cu/oxygen-enriched carbon battery confirmed the feasibility of the air-oxidation self-charging/electrical discharging mechanism for potential applications. This air-breathing chemical self-charge concept could facilitate the exploration of high-efficiency sustainable air self-charging devices.
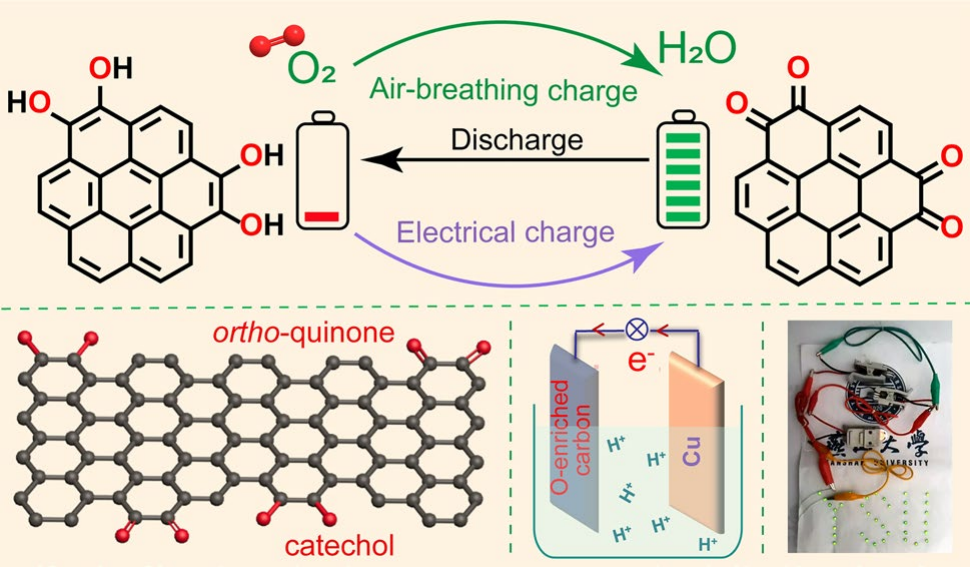

**Supplementary Information:**

The online version contains supplementary material available at 10.1007/s40820-023-01283-3.

## Introduction

Rechargeable electrochemical devices involving energy conversion and storage including batteries and supercapacitors have been widely investigated and used in various fields [1–9]. Self-charging concepts based on assembled energy conversion (e.g., solar cells, fuel cells, and triboelectric nanogenerators) and storage units (e.g., supercapacitors and batteries) [10–14], or integrated self-charging devices combining different energy conversion materials and storage materials in the electrodes [15, 16] exhibit a promising continual energy supply capacity through the utilization of environmental energy including solar energy [17, 18], mechanical energy based on the piezoelectric effect [19, 20], thermal energy based on the Soret effect [21], and chemical energy [22–24], especially in some specific human-related scenarios such as geological exploration, jungle operation, and individual combat. However, there are still two major issues: (1) Most of the environmental energy supply is limited by climate and meteorological conditions, while human motion energy utilization based on piezoelectric and triboelectric effects is restricted by workplace and sleep rules; these, hinder the popular application of these self-powered devices available at anytime and anywhere. (2) Most self-charging designs involve multiple energy harvesting, conversion and storage steps among different materials, which reduces the utilization efficiency of energy in the environment to a large extent.

An innovative design to achieve energy conversion and storage functions in single self-powered materials by utilizing natural sources, such as air, that is available at anytime and anywhere are high desirable, and this design could potentially ensure a continuous and controllable energy supply for the resulting self-powered devices with high energy efficiency. Based on the well-known ambient oxygen-triggered oxidizable characteristics of some metal oxides and organic conjugated structures [25], some researchers developed air self-charging aqueous zinc-ion batteries in single inorganic CaV_6_O_16_·3H_2_O, VO_2_, V_6_O_13_, or organic BQPH as the cathode. The chemical self-charging of these batteries could be achieved by the oxidation of the corresponding discharged cathode by oxygen molecules from the ambient environment without external power supply [26–29]. However, both the slow air oxidation-induced self-charging rate and low electrical conductivity of metal oxide and organic materials result in low content of active substance in cathode materials, largely inhibiting future applications of air self-charging power devices in areas lacking sufficient power supply networks. Compared with metal oxides and organic materials, carbon materials can exhibit outstanding electrical conductivity, excellent thermal and chemical stability, and heterogeneous atom-doping features, causing them to be especially suitable as electrode materials [30–33]. Heterogeneous atom doping to significantly promote the electrochemical activities of carbon materials has been widely accepted in the field of energy electrochemistry [34–37]. Therefore, in the self-charging field, the integration of reversible active heteroatom configurations with both spontaneous ambient oxygen-induced selective oxidation and subsequent electrochemical discharge characteristics to inert carbon materials are highly desirable, and proposing a chemical self-charging concept based on carbon materials is also imperative.

Based on aerobic autoxidation of catechol, similar to the electrochemical oxidation of catechol groups on carbon materials in electrical charge in our previous report [38], we propose the air-breathing chemical self-charge concept of a single oxygen-enriched carbon cathode material for a self-sustained power supply based on the aerobic autoxidation of the main catechol groups, similar to their oxidative transformation in the electrochemical charge process. The oxygen-enriched carbon material was synthesized through low-temperature carbonization and subsequent alkali activation of phenol–formaldehyde resin (PF). Subsequently, the assembled Cu/oxygen-enriched carbon device confirmed the feasibility of the air-oxidation self-charging/electrical discharging mechanism. Furthermore, compared with metal oxides and organic materials, oxygen-enriched carbon cathodes with good electrical conductivity exhibited a high active substance ratio of 85 wt%, which was extremely favorable for future applications of air self-charging devices. This conceptual air-breathing chargeable design based on a single oxygen-enriched carbon material with catechol groups could provide a practical concept to develop the sustainable high-efficiency self-powered devices.

## Experimental and Calculation

### Materials

Phenol (≥ 99.5%), formaldehyde (HCHO) (37 wt%), sodium hydroxide (NaOH, GR, 95%) and potassium hydroxide (KOH, GR, 95%) were purchased from Shanghai Macklin Biochemical Co., Ltd. Sulfuric acid (H_2_SO_4_, 98 wt%) and hydrochloric acid (HCl, 37 wt%) were supplied by Tianjin Kermel Chemical Reagent Co., Polyvinylidene difluoride (PVDF) was obtained from Guangdong Candlelight Amperex Technology Ltd., Commercial PF (2123) resin was purchased from Kafuman Chemical Co.

### Materials Preparation

Typically, phenol (0.1 g), aqueous HCHO solution (0.43 mL, 37 wt%), and aqueous NaOH solution (7 mL, 0.1 M) were dissolved in distilled water (72.57 mL). After stirring for 1 h, the above mixture was transferred to a 100 mL Teflon-lined stainless-steel autoclave and reacted at 160 °C for 4 h. The product was purified to neutrality several times with distilled water through a suction device and then dried at 60 °C for 12 h. Subsequently, the as-synthesized PF resin or commercial PF resin was heated to 475 °C with a ramp rate of 1 °C min^−1^, and then held for 4 h under pure nitrogen gas (99.999%) at a flow rate of 200 mL min^−1^. Finally, the powder mixture of the aforementioned pyrolyzed product and KOH at a weight ratio of 1/6 was heated at 475 °C for 8 h, followed by washing with an HCl solution and deionized water. After being dried at 60 °C for 12 h, the final activated product was obtained.

### Electrochemical Measurement

Electrochemical measurements and the air-breathing charging performance of all studies were tested in a three-electrode system with 1 M H_2_SO_4_ as the electrolyte. Typically, oxygen-enriched carbon materials derived from as-synthesized PF resin or commercial PF resin, acetylene carbon black, and PVDF binder at a weight ratio of 85:10:5 with 1-Methyl-2-pyrrolidinone (NMP) as the solvent were mixed to form slurry. The slurry was coated onto a 1 × 1 cm^2^ platinum net and dried at 120 °C under vacuum, and the mass loading of the active material as the working electrode was approximately 1 mg cm^−2^. Hg/Hg_2_Cl_2_ (SCE) and platinum plates (1 × 1 cm^2^) were used as the reference electrode and counter electrode, respectively.

For Cu-based self-sustained batteries, CR2032 coin cells were assembled with the as-obtained carbon paper-electrode (12 mm in diameter) as the cathode, Cu foil (12 mm in diameter) as the anode and NKK-MPF30AC-100 (16 mm in diameter) as the separator. The cathode was prepared by a conventional method. Briefly, the carbon paper-electrodes were prepared by coating the slurry onto carbon paper, and the slurry was prepared as mentioned above. The mass loading of a typical active material was ~ 3 mg. The cathode cap was predrilled with some holes to import oxygen. Before assembling the CR2032 coin cells, the carbon paper electrode was immersed in 1 M H_2_SO_4_ solution for 24 h in an air environment and the separator was wetted by 1 M H_2_SO_4_ electrolyte.

CV curves were measured at a scanning rate of 1–20 mV s^−1^ by a CHI 660E electrochemical workstation. The GCD tests were performed with a battery test system (Land CT3001A, China) in a voltage window from −0.15 to 0.85 V vs. SCE. The air-breathing charging performances were carried out by using a battery test system (Land CT3001A, China).

### Material Characterization

The microstructures of the samples were characterized via a HITACHI HT 7700 transmission electron microscope (TEM), with an accelerating voltage of 100 kV. HRTEM and EDS analyses were performed with a TALOS F200X G2 instrument operated at an acceleration voltage of 200 kV. The morphologies were acquired by a SUPRA 55 field emission scanning electron microscope (SEM) with an accelerating voltage of 200 kV. Raman spectra were recorded with a Raman microscope (HORIBA Xplora) using an Ar ion laser at an excitation wavelength of 532 nm. The powder XRD data were obtained via a RIGAKU X-ray diffractometer with Cu Kα radiation (l = 0.15418 nm) at 40 kV and the instrument was calibrated using the RIGAKU Silicon-640 as a standard sample before the measurement. The O K edge XAFS spectrum was acquired in the total electron yield (TEY) mode by BSRF 4B7B. X-ray photoelectron spectroscopy (XPS) analyses were performed with an ESCALAB 250 Xi spectrometer by using an Al Kα X-ray source. The pass energies for the XPS survey spectra and high-resolution XPS spectra test of all samples were 100.0 and 30.0 eV, respectively. All the high-resolution XPS spectra were fitted with the Lorentz/Gauss mixing ratios fixed at 0.8, sample charging was corrected by using the C 1*s* binding energy at 284.8 eV, and Shirley-type background subtraction was applied. As listed in Tables S1, S2, and S4, the FWHMs of O 1*s* were determined to be 1.7–1.9 eV. The ^13^C NMR spectrum was collected from the solid-state NMR 400 MHz, operating at 8.0 kHz.

## Results and Discussion

**Fig. 1:**
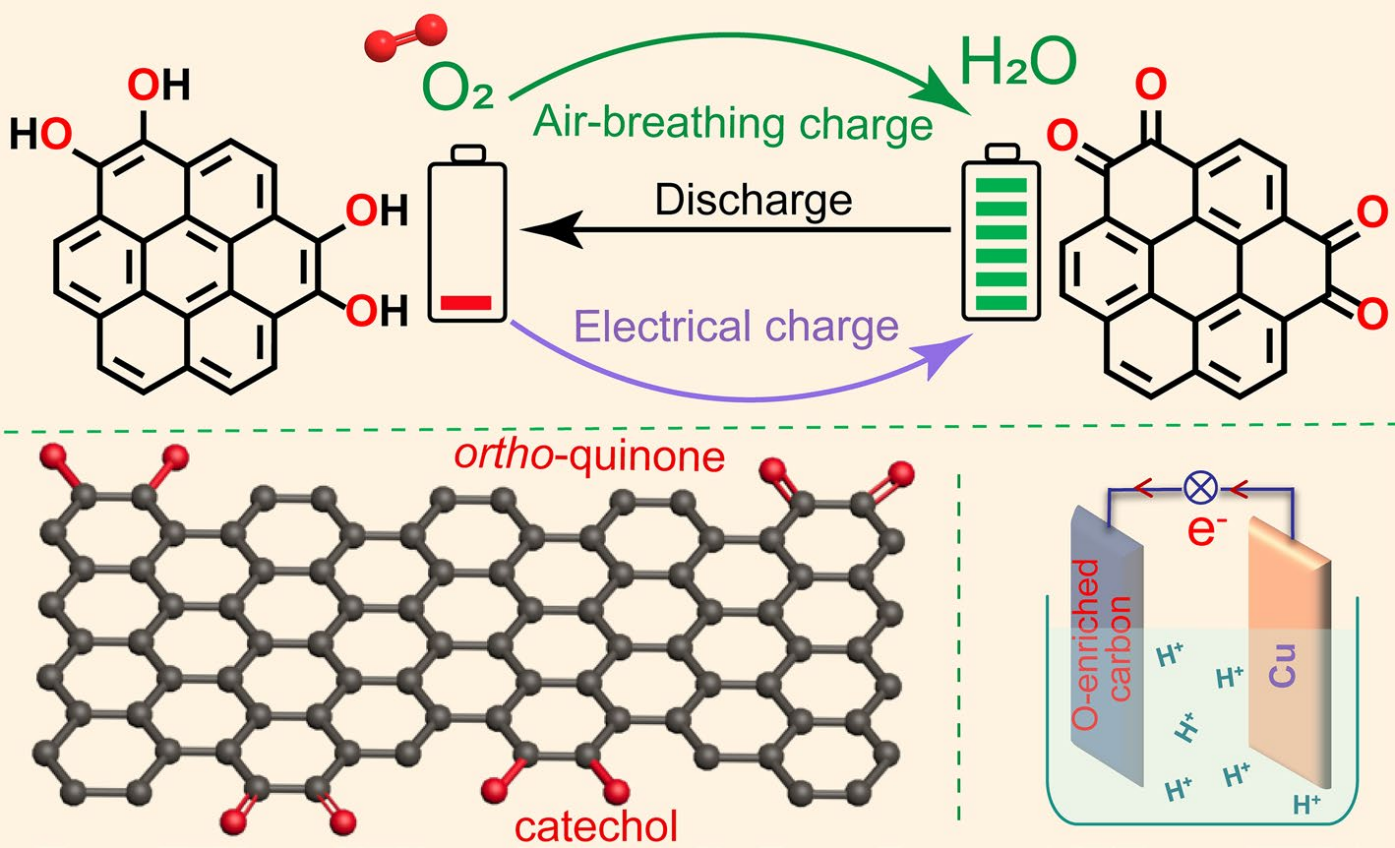
Air-breathing chemical self-charge concept of single oxygen-enriched carbon cathode material with catechol groups

Catechol could be transformed into *ortho*-quinone through aerobic autoxidation [39–41]; this reaction is similar to the electrochemical oxidation of catechol groups on oxygen-enriched carbon materials in the charging process [42, 43]. Thus, it could be reasonably inferred that the aerobic autoxidation of catechol groups on oxygen-enriched carbon materials could potentially achieve energy harvesting, conversion, and storage of oxygen-enriched carbon materials without an external power supply. Figure 1 shows the air-breathing chemical self-charge concept of catechol groups on the oxygen-enriched carbon material derived from phenol–formaldehyde (PF) resin as a single cathode material without any external power supply; this was different from the electrochemistry of these oxygen-containing groups with an external power supply. In response to external power stimuli, catechol groups on oxygen-enriched carbon materials in the cathode were oxidized to quinone-type groups along with the simultaneous loss of two protons and two electrons, thereby ensuring energy storage in the cathode. According to our previous report [38], the calculated redox potential of the *ortho*-quinone/catechol group couple on oxygen-enriched carbon material was approximately 0.60 V versus standard hydrogen electrode (SHE) in the acidic medium; this value was far lower than the redox potential of the O_2_/H_2_O couple in the acidic medium (1.23 V vs. SHE). Thus, the spontaneous oxidation of the catechol groups on the oxygen-enriched carbon material to *ortho*-quinone groups was thermodynamically favorable. Therefore, designing a single air-breathing chemical self-charged oxygen-enriched carbon material as the cathode to achieve energy harvesting, conversion and storage was theoretically possible for self-sustained power supply. In addition, the performance of a complete secondary battery is generally dependent on two redox couples with different redox potentials in the cathode and anode. Based on the redox reaction of the oxygen-enriched carbon materials as the positive electrode involving proton and electron transfer in the air-breathing charge/discharge cycles, an acid-resistant material with low redox potential needs be chosen as the negative electrode. The standard redox potential of Cu^2+^/Cu(0) couples (0.3419 V) is higher than that of H^+^/H_2_ couples (0 V) and lower than the calculated counterpart of the *ortho*-quinone/catechol group couple on oxygen-enriched carbon material (0.60 V); therefore, catechol group-enriched carbon materials as the cathode and the copper anode separated by an acidic electrolyte could be theoretically assembled into air-breathing self-charge devices.

### Proof of the Air-Breathing Self-charge Concept Based on Oxygen-Enriched Carbon Materials

**Fig. 2:**
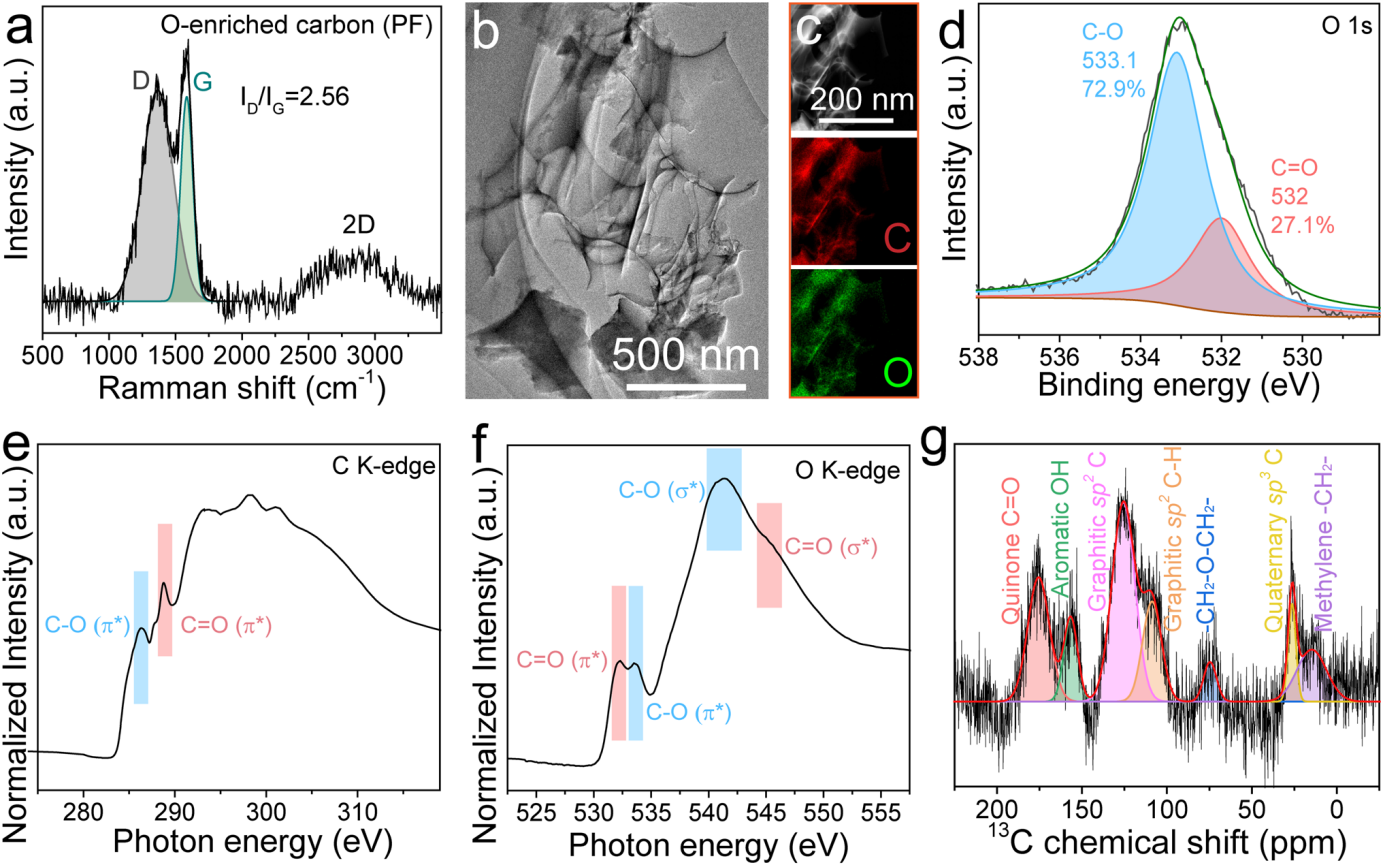
Structural characterization of oxygen-enriched carbon (PF) material. **a** Raman spectrum. **b** TEM, **c** HADF-STEM images and C, O elemental mapping. **d** High-resolution XPS spectrum of O 1*s*. **e, f** High-resolution NEXAFS spectra of C and O K-edge. **g** Solid-state ^13^C NMR spectrum

To verify our air-breathing chemical self-charge device concept, a catechol group-enriched carbon material was initially synthesized through low-temperature carbonization and subsequent alkali activation of phenol–formaldehyde resin. Its Raman spectrum (Fig. 2a) showed a typical feature of *sp*^2^-hybridized amorphous carbon material with abundant defects, according to the broad D band at 1356 cm^−1^ and G band at 1585 cm^−1^ corresponding to the breathing mode of the A1g symmetry and in-plane bond-stretching of *sp*^2^ carbon atoms, respectively [44, 45]. Its XRD pattern (Fig. S1a) also showed the characteristics of amorphous carbon materials with broadened (002) and (100)/(101) diffraction peaks at 23.9° and 43.3°, respectively, analogous to the features of graphite [46]. In addition, the SEM (Fig. S1b), TEM (Fig. 2b), and HRTEM (Fig. S1c) images further verified the amorphous feature of the oxygen-enriched carbon (PF) material in the form of nanosheets with long-range disorder and local short-range order. The EDS elemental mapping images combined with HADF-STEM images (Fig. 2c) showed a uniform distribution of oxygen species in the carbon framework of the oxygen-enriched carbon (PF) material. Its XPS survey spectrum (Fig. S2) further verified the existence of the abundant oxygen species in the carbon materials. Its high-resolution O 1*s* XPS spectrum (Fig. 2d, Table S1) could be deconvoluted into two different peaks at 533.1 and 532.0 eV, corresponding to 72.9% hydroxyl/ether (C–OH/C–O–C) and 27.1% carbonyl (C=O) groups, respectively. In addition, the C K-edge NEXAFS spectrum (Fig. 2e) showed the well-resolved C 1*s*-π* transition of C–O at 286.3 eV and C=O at 288.8 eV. The O K-edge NEXAFS spectrum (Fig. 2f) also showed the evident O 1*s*-π* and 1*s*-σ* transitions of C=O (532.3 and 545.0 eV, respectively) and C–O (533.6 and 541.4 eV, respectively). To precisely confirm oxygen-enriched groups on the carbon material, solid-state ^13^C NMR spectroscopy of the oxygen-enriched carbon (PF) material was performed (Fig. 2g). Two peaks at 125.7 ppm and 109.0 ppm corresponded to graphitic *sp*^2^-hybridized C and C–H, respectively. A smaller peak at 26.3 ppm, a broader one at 14.7 ppm, and an independent one at 74.5 ppm was assigned to quaternary *sp*^3^-hybridized C, methylene –CH_2_–, and –CH_2_–O–CH_2_–, respectively. Moreover, two strong peaks at 176.0 and 156.5 ppm effectively matched with the characteristics of quinone C=O and aromatic OH, respectively [47], which confirmed the existence of catechol groups in the oxygen-enriched carbon (PF) material.

**Fig. 3:**
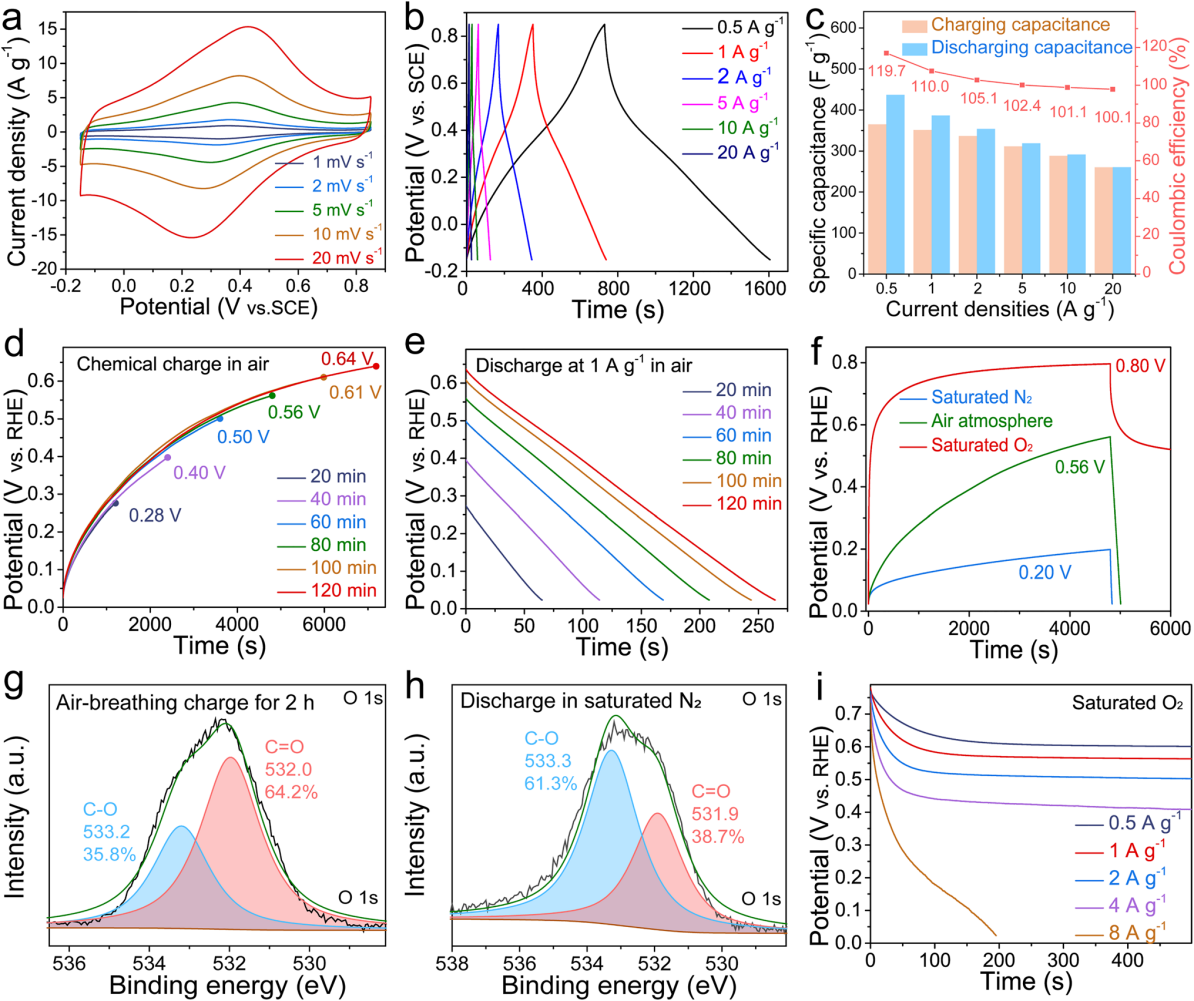
Electrochemical performance evaluation of oxygen-enriched carbon (PF) material. **a** CV tests. **b** Galvanostatic charge/discharge curves under air atmosphere. **c** The corresponding specific charge/discharge capacitance values and coulombic efficiency calculated through the GCD curves in Fig. 3b. **d** Time-dependent chemical charge potential curves at different time under air atmosphere. **e** The corresponding time-dependent galvanostatic discharge behavior at 1 A g^−1^ after being chemically charged in Fig. 3d. **f** Chemical charge curves and galvanostatic discharge curves at 1 A g^−1^ in saturated N_2_, air, and saturated O_2_ atmosphere for 80 min. High-resolution O 1*s* XPS spectra of oxygen-enriched carbon (PF) material **g** after being chemically charged for 120 min in air, and **h** after being discharged at 0.1 A g^−1^ in saturated N_2_. **i** Galvanostatic discharge curves at different current density after being oxidized for 80 min in saturated O_2_. All of the electrochemical tests mentioned above were carried out in a three-electrode system with 1 M H_2_SO_4_ aqueous solution

The CV curves of the oxygen-enriched carbon (PF) electrode in air at different scan rates (1–20 mV s^−1^) with a pair of symmetrical peaks (0.3–0.4 V vs. SCE) (Fig. 3a) and the symmetrical GCD curves in air at various current densities with distorted linear shapes (Fig. 3b) in 1 M H_2_SO_4_ aqueous solution exhibited reversible Faraday redox reactions corresponding to the conversion of the catechol groups into the *ortho*-quinone groups [42, 43], which further confirmed the existence of catechol groups in the oxygen-enriched carbon (PF) material. An intriguing and counterintuitive phenomenon was clearly observed in the charge/discharge process of the oxygen-enriched carbon (PF) material (Fig. 3c) in air. The discharge capacitances were higher than the corresponding charge capacitances; thus, the coulombic efficiencies were higher than 100% at current densities from 0.5 to 20 A g^−1^. In addition, the gap between the discharge capacitance and charge capacitance and coulombic efficiency gradually decreased with increasing current density. In order to elucidate the cause behind the coulombic efficiencies of oxygen-enriched carbon (PF) material in air higher than 100%, controlled electrochemical performances of the oxygen-enriched carbon (PF) electrode in 1 M H_2_SO_4_ solution in different atmospheres including saturated N_2_ and O_2_ were also evaluated (Fig. S3 and Note S1). Different from the coulombic efficiencies of oxygen-enriched carbon (PF) material at current densities from 0.5 to 20 A g^−1^ in air higher than 100%, those in saturated N_2_ were equivalent to or slightly lower 100% (Fig. S3c). Interestingly, the coulombic efficiencies of oxygen-enriched carbon (PF) material in saturated O_2_ were far higher than 100% and also higher than those in air at current densities from 0.5 to 20 A g^−1^ (Fig. S3f). Based on these results, there was an unknown independent O_2_-related charging mechanism accompanied by power charging. Since the calculated redox potential of *ortho*-quinone/catechol group couples on our oxygen-enriched carbon (PF) material (approximately 0.60 V vs. SHE) in Fig. 3a is far lower than the redox potential of the O_2_/H_2_O couple in the acidic medium (1.23 V vs. SHE), catechol groups of oxygen-enriched carbon (PF) material were likely spontaneously oxidized by oxygen to corresponding *ortho*-quinone groups; this process was similar to the Faradic reactions of the catechol groups on an oxygen-enriched carbon (PF) cathode in the charging process in air atmosphere. Therefore, the harvesting, conversion, and storage of chemical energy derived from oxygen accompanied by power charging was achieved.

To reliably verify our air-breathing chemical self-charge hypothesis, we initially performed time-dependent air-breathing charge (Fig. 3d) and galvanostatic discharge at 1 A g^−1^ (Fig. 3e) for the oxygen-enriched carbon (PF) material in a three-electrode system in 1 M H_2_SO_4_ aqueous solution in an air atmosphere to exclude the effect of power charging. The potential of the oxygen-enriched carbon (PF) electrode gradually increased from 0.28 to 0.64 V vs. RHE with prolonged air-breathing charge time from 20 to 120 min. The corresponding discharge capacitances at 1 A g^−1^ were calculated to be 65, 114, 168, 208, 244, and 264 F g^−1^ for 20, 40, 60, 80, 100, and 120 min, respectively (Table S2). Evidently, the discharge capacitance of the oxygen-enriched carbon (PF) electrode was positively dependent on the air-breathing charge time, which proves air-breathing charge mechanism to some content.

To further elucidate the air-breathing chemical self-charge effect, chemical charge and galvanostatic discharge at 1 A g^−1^ of an oxygen-enriched carbon (PF) electrode in 1 M H_2_SO_4_ solution in different atmospheres including saturated N_2_, air, and saturated O_2_ were performed. After chemical charging for 80 min, the potentials of the oxygen-enriched carbon (PF) electrode in the above atmospheres increased to 0.20, 0.56, and 0.80 V vs. RHE, respectively, highly consistent with the oxygen content in saturated N_2_, air, and saturated O_2_ (Fig. 3f), which further confirmed that oxygen-induced oxidation of oxygen-enriched carbon (PF) material played a crucial role in the chemical charging process. To further clarify the air-breathing charging mechanism driven by oxygen, the pristine oxygen-enriched carbon (PF) materials after air-breathing charge for 120 min and galvanostatic discharge in saturated N_2_ at 0.1 A g^−1^ were recovered to monitor the mutual conversion of the oxygen-enriched groups in chemical charge and subsequent galvanostatic discharge using XPS (Fig. 3g, h, Table S3). According to high-resolution O 1*s* XPS spectra of oxygen-enriched carbon (PF) material in different states, after air-breathing charge and subsequent galvanostatic discharge in saturated N_2_ at 0.1 A g^−1^, the content of hydroxyl groups initially decreased initially from 72.9 at% (~ 533.1 eV, Fig. 2d) to 35.8 at% (~ 533.2 eV, Fig. 3g) and then increased to 61.3 at% (~ 533.3 eV, Fig. 3h); the content of carbonyl groups increased from 27.1 at% (~ 532.0 eV, Fig. 2d) to 64.2 at% (~ 532.0 eV, Fig. 3g) and then decreased to 38.7 at% (~ 531.9 eV, Fig. 3h). These results were potentially attributed to the O_2_-triggered oxidation of catechol to *ortho*-quinone and the subsequent galvanostatic electroreduction reaction of the quinone groups for the pristine oxygen-enriched carbon (PF) material; this process was highly similar to the reversible conversion between quinone and the catechol groups on carbon materials in the galvanostatic charge/discharge process [38]. Thus, this further confirmed that the O_2_-triggered oxidation of catechol to *ortho*-quinone accounted for the air-breathing charge mechanism in oxygen-enriched carbon (PF) material. In addition to the positive dependence of the potential on the oxygen content in different atmospheres, a unique persistent potential plateau in the curve of galvanostatic discharge at 1 A g^−1^ in saturated O_2_ was observed; this result was, completely different from galvanostatic discharge behaviors of previously reported carbon materials with pseudocapacitive activity in air [48, 49]. To investigate the cause behind this unique persistent potential plateau in the galvanostatic discharge curve, galvanostatic discharge behaviors at various current densities from 0.5 to 8 A g^−1^ after being oxidized for 80 min in saturated O_2_ were evaluated (Fig. 3i). With prolonged galvanostatic discharge time, the potential exhibited an initial reduction tendency and a subsequent persistent potential plateau in the discharge current density ranging from 0.5 to 4 A g^−1^. This unique discharge phenomenon possibly stemmed from the following cause. In the initial discharging stage, few catechol groups were available for O_2_ oxidation, leading to lower O_2_-breathing charge rate than the spontaneous galvanostatic discharge rate. The potential gradually decreased with prolonged discharge time; thus, the energy was gradually released and more *ortho*-quinone groups were electrically reduced to their corresponding catechol groups available for O_2_ oxidation, thereby gradually increasing the O_2_-breathing charge rate. When the increased O_2_-breathing charge rate was equivalent to the galvanostatic discharge rate, the constant potential no longer changed over time. Based on the above results, the oxygen-enriched carbon (PF) cathode could achieve chemical energy harvesting from O_2_, conversion and storage in the form of electrical energy even in the galvanostatic discharge process. With an increase in the galvanostatic discharge current density from 0.5 to 4 A g^−1^, only the further potential reduction could reduce more *ortho*-quinone groups to the corresponding catechol groups available for O_2_ oxidation to increase the O_2_-breathing charge rate up to the corresponding galvanostatic discharge rate. The values of the plateau potentials at 0.5, 1, 2, and 4 A g^−1^ were also determined to be approximately 0.60, 0.56, 0.50, and 0.41 V, respectively. Therefore, the values of the plateau potential were negatively correlated with the galvanostatic discharge current density. When the galvanostatic discharge current density reached 8 A g^−1^, the discharge potential gradually decreased, and there was no potential plateau with prolonged discharge time, which was attributed to the O_2_-breathing charge rate being lower than the galvanostatic discharge rate throughout the discharge process. Our air-breathing chargeable mechanism based on an oxygen-enriched carbon (PF) cathode provides an innovative charging mechanism using air available at any time and at any site, especially in some areas without sufficient power supply networks. Considering that the air-breathing charging rate of the oxygen-enriched carbon (PF) cathode is generally lower than the galvanostatic charging rate, the air-breathing and galvanostatic hybrid charge performance of the oxygen-enriched carbon (PF) cathode is significant for its future application, especially frequently at sites with and without sufficient power supply networks. As shown in Fig. S4, after air-breathing charge to 0.56 V for 80 min in air and subsequent galvanostatic discharge at 1 A g^−1^, the next mixed air-breathing charge to 0.55 V vs. RHE for 80 min in air and galvanostatic charge at 1 A g^−1^ to 1.07 V vs. RHE could be achieved. Then, galvanostatic discharge/charge at 1 A g^−1^ was also achieved. These results demonstrated that the oxygen-enriched carbon (PF) cathode could efficiently achieve the air-breathing chemical and galvanostatic hybrid charge mode. To further evaluate the stability of the oxygen-enriched carbon (PF) material in the air-breathing chemical charge and galvanostatic discharge process, the potential of the oxygen-enriched carbon (PF) electrode after 1,200 cycles of air-breathing chemical charge for 20 min and galvanostatic discharge at 1 A g^−1^ still maintained the initial potential (~ 0.21 V vs. RHE) (Fig. S5). Therefore, the air-breathing chargeable oxygen-enriched carbon (PF) material could be expected to exhibit high stability in future practical applications.

**Fig. 4:**
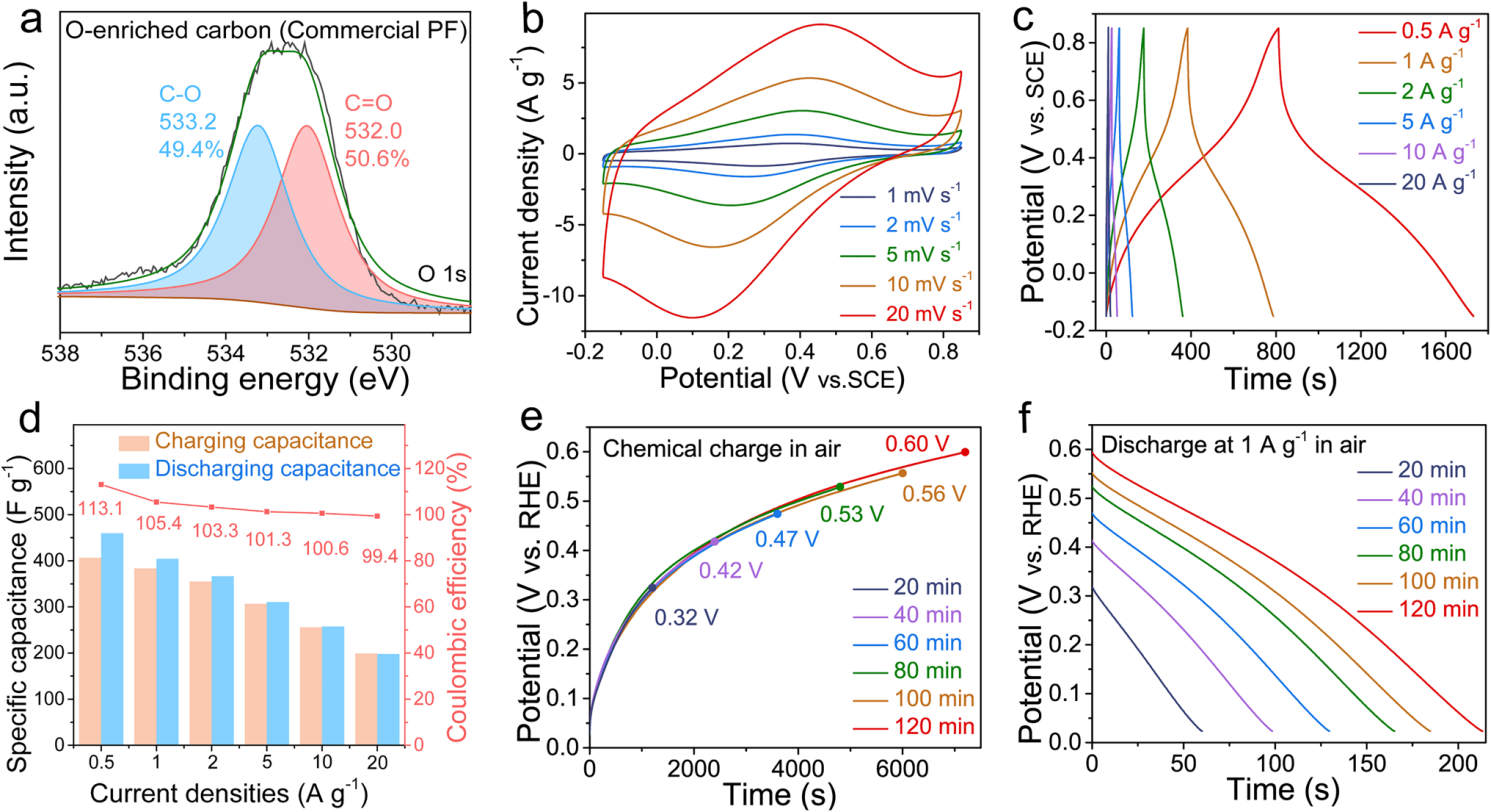
Electrochemical performance evaluation of oxygen-enriched carbon (Commercial PF) material. **a** High-resolution O 1*s* XPS spectrum of oxygen-enriched carbon (Commercial PF) material. **b** CV test and **c** GCD curves under air atmosphere. **d** The corresponding specific charge/discharge capacitance values and coulombic efficiency calculated by the GCD curves in Fig. 4c at different current densities. **e** Chemical charging potential profile curves were measured at different chemical chargeable time under air atmosphere. **f** The corresponding galvanostatic discharge curves at 1 A g^−1^ in Fig. 4e

To verify the air-breathing chemical self-charge mechanism, another oxygen-enriched carbon material was also obtained through the low-temperature carbonization and subsequent alkali activation of commercial phenol–formaldehyde (Commercial PF) resin similar to that of as-synthesized PF resin (Figs. S6, S7, and Note S2). As shown in Fig. 4a and Table S4, its high-resolution O 1*s* XPS spectrum also showed the C–OH/C–O–C and C=O features at 533.2 and 532.0 eV, respectively. Its CV curve (Fig. 4b) and GCD curve (Fig. 4c) also exhibited typical pseudocapacitive behavior, corresponding mainly to the conversion of the catechol groups into *ortho*-quinone groups. The coulombic efficiencies were higher than 100% at current densities from 0.5 to 10 A g^−1^, which also meant the existence of an extra chemical charge mechanism (Fig. 4d). The potential of the oxygen-enriched carbon (Commercial PF) electrode increased from 0.32 to 0.60 V vs. RHE in air along with the prolonged air-breathing charging time from 20 to 120 min, and the corresponding discharge capacitance also increased from 60 to 213 F g^−1^ (Fig. 4e, f, Table S5) at 1 A g^−1^; this process was similar to the air-breathing charge/galvanostatic discharge process of the oxygen-enriched carbon (PF) material (Fig. 3d, e). The above results further confirmed our air-breathing charge mechanism.

### Air-Breathing Chemical Self-charge Potential Application of the Oxygen-Enriched Carbon (PF) Cathode

**Fig. 5:**
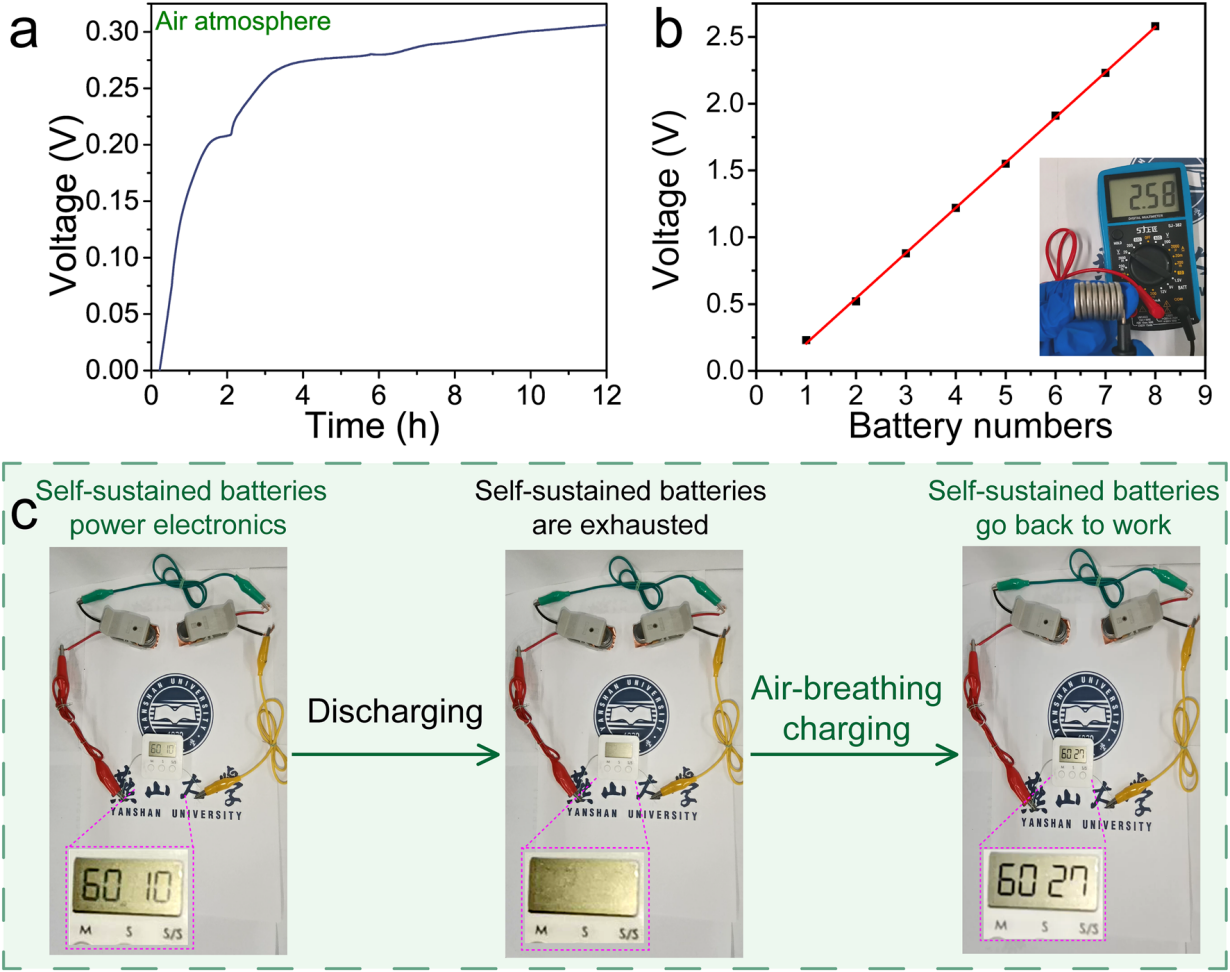
Demonstration of the self-sustained batteries. **a** Voltage–time curve of the self-sustained battery under air atmosphere. The voltage of the self-sustained battery was measured after galvanostatic discharge at 1 A g^−1^ to ~ 0 V vs RHE). **b** The number-dependent potential curve of self-sustained battery. **c** Electronics timer powered by six self-sustained batteries at different states

Considering transfer and transport kinetics of electron and electrolyte ions crucial for the application of electrode materials, EIS analysis of the oxygen-enriched carbon (PF) material was performed. Nyquist plots and corresponding fitted equivalent circuit were shown in Fig. S8 and insets. A nearly straight line at low frequency region demonstrates ideal capacitive behavior (Fig. S8a), the low equivalent series resistance (~ 0.90 Ω) reveals good electron and ion transport ability (Fig. S8a inset), and the small charge transfer resistance (~ 0.33 Ω) highly facilitates electrochemical redox reaction at the interface between the electrode material and electrolyte (Fig. S8b). The results mentioned above demonstrates that the oxygen-enriched carbon (PF) material could be used as ideal electrode material. To further explore the potential application of the air-breathing charging mechanism of the oxygen-enriched carbon materials, an oxygen-enriched carbon (PF) material coated on carbon paper as the cathode and Cu foil as the anode was assembled into a self-sustained battery. As shown in Fig. 5a, with the time of the cathode exposed to an air atmosphere prolonged to 12 h, the voltage of the self-sustained battery gradually increased to ~ 0.31 V vs. RHE, which was consistent with the potential difference between oxygen-enriched carbon (PF) and Cu. In addition, the voltage was positively dependent on the number of self-sustained batteries when multiple batteries were serially connected (Fig. 5b), which ensured the power supply to different electronic devices. As expected, six serial self-sustained batteries could continuously power a timer, and the subsequent air-breathing charge of these exhausted batteries supplemented with only a small amount of 1 M H_2_SO_4_ solution supplemented and without any external power supply could make the sustained batteries to work again (Fig. 5c). The results mentioned above indicate that developing a simple air self-charging device with energy collection, conversion and storage based on the air-breathing charge mechanism of catechol groups on oxygen-enriched carbon materials could be promising in the portable electronics applications available at any time, and at anywhere without the need of an external power supply.

## Conclusions

In summary, we proposed an air-breathing self-charge concept based on catechol groups on oxygen-enriched carbon materials. The potential of the oxygen-enriched carbon (PF) electrode gradually increased from 0.28 to 0.64 V vs. RHE with prolonged air-breathing charge time from 20 to 120 min, which was attributed to air oxidation of the catechol groups on the oxygen-enriched carbon material to *ortho*-quinone groups. Based on the air self-charging mechanism on a single carbon material, the assembled Cu/oxygen-enriched carbon battery showed its practical application. Our air-breathing charge concept facilitates the design of rapid air self-charging devices based on oxygen-enriched carbon materials, particularly in some harsh environments unavailable for sufficient external power supply.

## Supplementary Information

Below is the link to the electronic supplementary material.

Supplementary file1 (PDF 874 KB)
