# Supplementary material for: Proof of Aerobically Autoxidized Self-Charge Concept Based on Single Catechol-Enriched Carbon Cathode Material
Source: Nanomicro Lett. 2023 Dec 20;16:62. doi: 10.1007/s40820-023-01283-3 (PMC10733265; doi:10.1007/s40820-023-01283-3)
Supplement: Supplementary file 1 — Supplementary file1 (PDF 874 KB) [file 40820_2023_1283_MOESM1_ESM.pdf]

Supporting Information for

# Proof of Aerobically Autoxidized Self-Charge Concept Based on Single Catechol-Enriched Carbon Cathode Material

Junyan Wang[1], Wanchun Guo[1,*], Kesong Tian[1,*], Xinta Li[1], Xinyu Wang[1], Panhua Li[1], Yu Zhang[1], Bosen Zhang[1], Biao Zhang[2], Shuhu Liu[3], Xueai Li[1], Zhaopeng Xu[1], Junjie Xu[4], Haiyan Wang[1,*], Yanglong Hou[2,*]

[1] State Key Laboratory of Metastable Materials Science and Technology, Hebei Key Laboratory of Heavy Metal Deep-Remediation in Water and Resource Reuse, School of Environmental and Chemical Engineering, Yanshan University, 066004 Qinhuangdao, P. R. China

[2] Beijing Key Laboratory for Magnetoelectric Materials and Devices, Beijing Innovation Centre for Engineering Science and Advanced Technology, School of Materials Science and Engineering, Peking University, 100871 Beijing, P. R. China

[3] Institute of High Energy Physics, Chinese Academy of Sciences, 100049 Beijing, P. R. China

[4] Xi'an Rare Metal Materials Institute Co., Ltd, 710016 Xi'an, P. R. China

*Corresponding authors. E-mail: wc-g@ysu.edu.cn (Wanchun Guo); tiankesong@ysu.edu.cn (Kesong Tian); hywang@ysu.edu.cn (Haiyan Wang); hou@pku.edu.cn (Yanglong Hou)

## Supplementary Figures

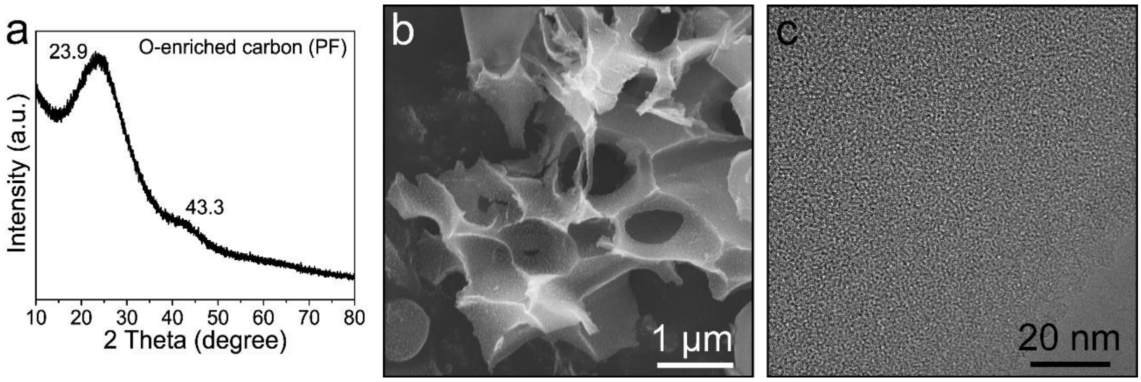


**Fig. S1** Structural characterization of oxygen-enriched carbon (PF) material. **a** XRD pattern, **b** SEM, **c** HRTEM images

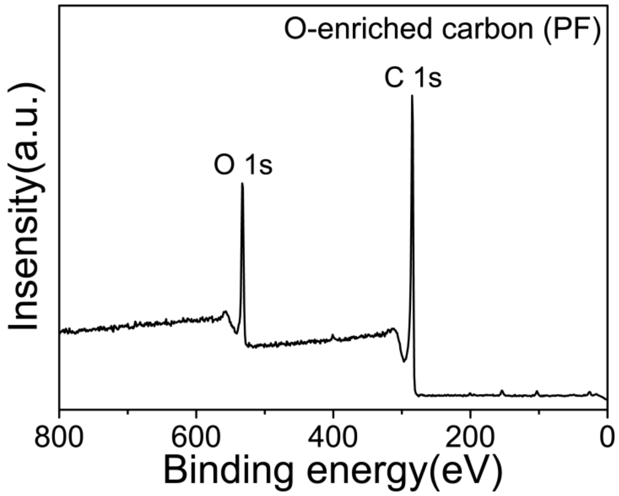


**Fig. S2** XPS survey spectrum of oxygen-enriched carbon (PF) material

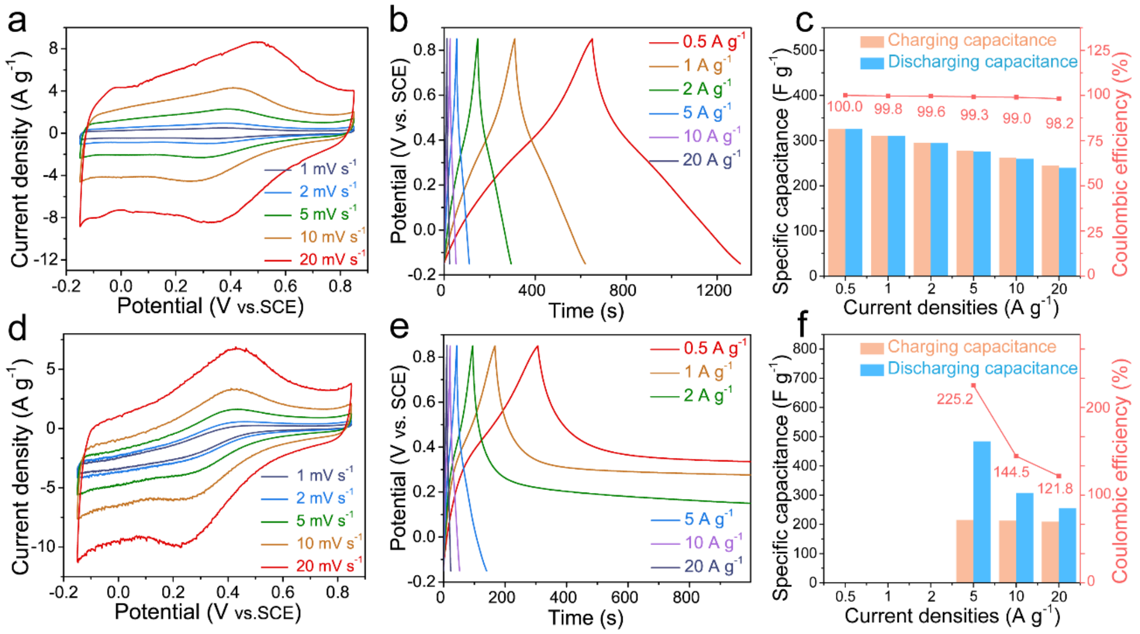


**Fig. S3** Electrochemical performance evaluation of oxygen-enriched carbon (PF) material. **a** CV tests under saturated $N_2$ atmosphere. **b** Galvanostatic charge/discharge curves under saturated $N_2$ atmosphere. **c** The corresponding specific charge/discharge capacitance values and coulombic efficiency calculated through the GCD curves in Fig. S3b. **d** CV tests under saturated $O_2$ atmosphere. **e** Galvanostatic charge/discharge curves under saturated $O_2$ atmosphere. **f** The corresponding specific charge/discharge capacitance values and coulombic efficiency calculated through the GCD curves in Fig. S3e

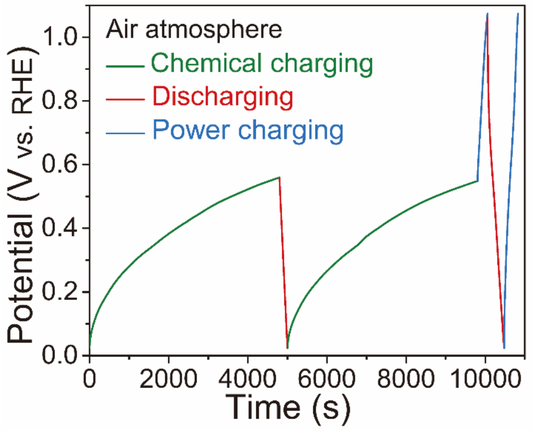


**Fig. S4** Charge/discharge behavior of oxygen-enriched carbon (PF) material at chemical or/and galvanostatic charge hybrid modes in air atmosphere in a three-electrode system with 1 M $H_2SO_4$ aqueous solution

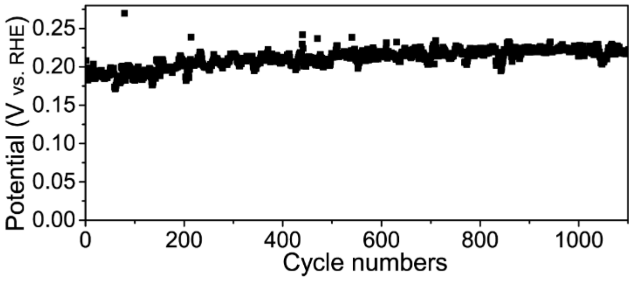


**Fig. S5** Electrochemical stability of oxygen-enriched (PF) material. In every cycle, the oxygen-enriched carbon (PF) electrode was chemically charged at air atmosphere for 20 min to measure the potential and subsequently discharged at 1 A $g^{-1}$ in a three-electrode system with 1 M $H_2SO_4$ aqueous solution as the electrolyte

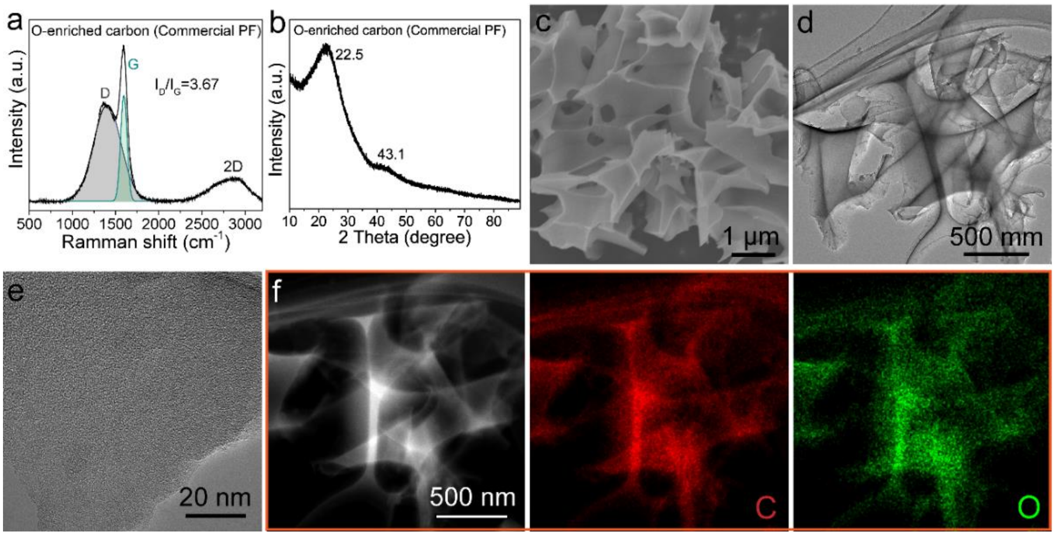


**Fig. S6** Characterization of oxygen-enriched carbon (Commercial PF) material. **a** Raman spectrum. **b** XRD pattern. **c** SEM. **d** TEM. **e** HRTEM. **f** HADF-STEM images and the elemental mapping images of carbon and oxygen

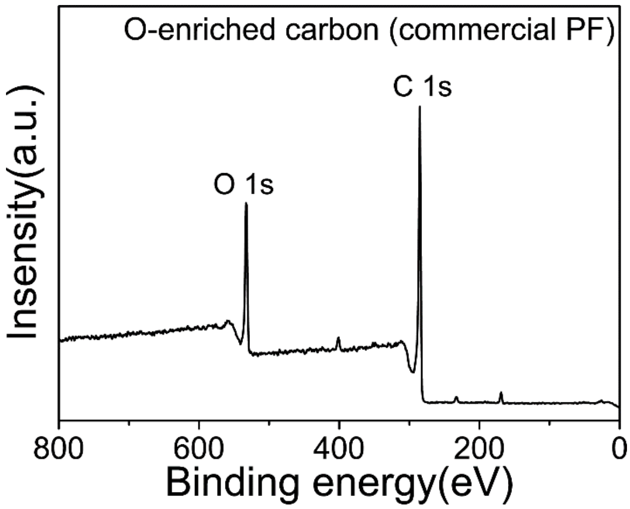


**Fig. S7** XPS survey spectrum of oxygen-enriched carbon (Commercial PF) material

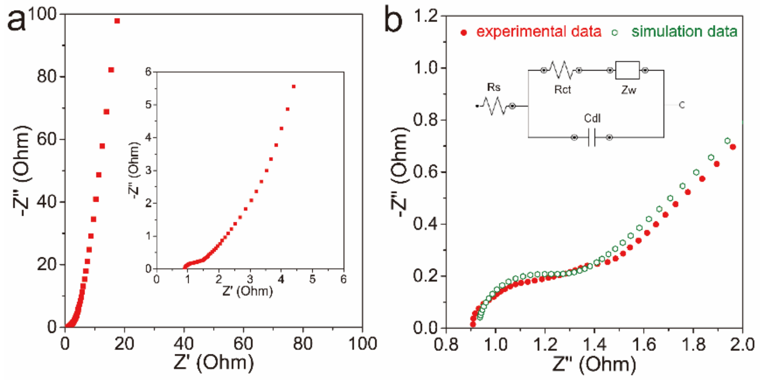


**Fig. S8** Electrochemical impedance spectroscopy (EIS) for oxygen-enriched carbon (PF) cathode. **a** Complex-plane plots of EIS in frequency ranged from 0.01 to 100,000 Hz in 1.0 M $H_2SO_4$. The inset shows the corresponding details at high-frequency ranges. **b** Nyquist plots (red dots) and Nyquist plot simulation (green circles) of EIS. The inset

**Table S1** Binding energies and FWHMs for O 1s XPS spectrum of oxygen-enriched carbon (PF) material in Fig. 2

| Sample | -C=O B.E. (eV) | -C=O FWHM (eV) | -C-O B.E. (eV) | -C-O FWHM (eV) |
|---|---|---|---|---|
| O-enriched carbon (PF) | 532.0 | 1.7 | 533.1 | 1.7 |

**Table S2** The capacitance of air-breathing chargeable system of oxygen-enriched carbon (PF) material in three-electrode system at 1 A $g^{-1}$ in 1.0 M $H_2SO_4$ electrolyte.

| Oxidized time (min) | 20 | 40 | 60 | 80 | 100 | 120 |
|---|---|---|---|---|---|---|
| Capacitance (F $g^{-1}$) | 65 | 114 | 168 | 208 | 244 | 264 |

**Table S3** Binding energies and FWHMs for O 1s XPS spectrum of oxygen-enriched carbon (PF) material in Fig. 3

| Sample | -C=O B.E. (eV) | -C=O FWHM (eV) | -C-O B.E. (eV) | -C-O FWHM (eV) |
|---|---|---|---|---|
| Air-breathing charging for 2 h | 532.0 | 1.8 | 533.2 | 1.7 |
| Discharging in Saturated $N_2$ | 531.9 | 1.8 | 533.3 | 1.9 |

**Table S4** Binding energies and FWHM for O 1s of oxygen-enriched carbon (Commercial PF) material in Fig. 4

| Sample | -C=O B.E. (eV) | -C=O FWHM (eV) | -C-O B.E. (eV) | -C-O FWHM (eV) |
|---|---|---|---|---|
| O-enriched carbon (Commercial PF) | 532.0 | 1.8 | 533.2 | 1.8 |

**Table S5** The capacitance of air-breathing chargeable system of oxygen-enriched carbon (Commercial PF) material in three-electrode system at 1 A $g^{-1}$ in 1.0 M $H_2SO_4$ electrolyte.

| Oxidized time (min) | 20 | 40 | 60 | 80 | 100 | 120 |
|---|---|---|---|---|---|---|
| Capacitance (F $g^{-1}$) | 60 | 99 | 130 | 165 | 185 | 213 |

## Supplementary Results and Discussion

### Note S1 Electrochemical performance evaluation of oxygen-enriched carbon (PF) material in saturated $N_2$ and $O_2$

In order to elucidate the cause behind the coulombic efficiencies of oxygen-enriched carbon (PF) material in air higher than 100% at current densities from 0.5 to 20 A $g^{-1}$, electrochemical performances of the oxygen-enriched carbon (PF) electrode in 1 M $H_2SO_4$ solution in different atmospheres including saturated $N_2$ and $O_2$ were evaluated. (1) The CV curves of the oxygen-enriched carbon (PF) electrode in saturated $N_2$ at different scan rates (1-20 mV $s^{-1}$) with a pair of symmetrical peaks (0.3-0.4 V vs. SCE) (Fig. S3a) and the symmetrical GCD curves at various current densities with distorted linear shapes (Fig. S3b) in 1 M $H_2SO_4$ aqueous solution exhibited reversible Faraday redox reactions corresponding to the mutual conversion between catechol and *ortho*-quinone groups. Different from the coulombic efficiencies of oxygen-enriched carbon (PF) material at current densities from 0.5 to 20 A $g^{-1}$ in air higher than 100%, those in saturated $N_2$ were equivalent to or slightly lower 100% (Fig. S3c). (2) The CV curves of the oxygen-enriched carbon (PF) electrode in saturated $O_2$ at different scan rates (1-20 mV $s^{-1}$) with a pair of symmetrical peaks (0.3-0.4 V vs. SCE) (Fig. S3d) in 1 M $H_2SO_4$ aqueous solution exhibited reversible Faraday redox reactions corresponding to the mutual conversion

between catechol and *ortho*-quinone groups. According to GCD curves in saturated $O_2$ at various current densities from 0.5 to 20 A $g^{-1}$ (Fig. S3e), the discharge capacitances were far higher than the corresponding charge capacitances; thus, the coulombic efficiencies were far higher than 100% at current densities from 0.5 to 20 A $g^{-1}$ (Fig. S3f).

**Note S2 Structural Characterization of oxygen-enriched carbon (Commercial PF) material**

Raman spectrum of oxygen-enriched carbon (Commercial PF) material (Fig. S6a) shows a typical feature of $sp^2$-hybridized amorphous carbon materials with abundant defects, according to broad D band at 1398 $cm^{-1}$ and G band at 1594 $cm^{-1}$ corresponding to the breathing mode of the A1g symmetry and in-plane bond-stretching of $sp^2$ carbon atoms, respectively. Its X-ray diffraction (XRD) pattern (Fig. S6b) also provides another evidence of the characteristic of amorphous carbon materials with broadened (002) and (100)/(101) diffraction peaks at 22.5° and 43.1° analogous to the features of graphite. In addition, the scanning electron microscopy (SEM) (Fig. S6c), transmission electron microscopy (TEM) (Fig. S6d), and high-resolution transmission electron microscopy (HRTEM) (Fig. S6e) images further verify the amorphous feature of oxygen-enriched carbon (Commercial PF) material in the form of the nanosheet with the long-range disorder and local short-range order structure. Energy dispersive X-ray spectroscopy (EDS) elemental mapping images combined with HADF-STEM image (Fig. S6f) reveal a uniform distribution of oxygen species into carbon framework for oxygen-enriched carbon (Commercial PF) material. X-ray photoelectronic spectroscopy (XPS) survey spectrum of oxygen-enriched carbon (Commercial PF) material (Fig. S7) further verifies the existence of abundant oxygen species in carbon materials.
